# Design and Simulation of Production and Maintenance Management Applying the Viable System Model: The Case of an OEM Plant

**DOI:** 10.3390/ma11081346

**Published:** 2018-08-03

**Authors:** Sergio Gallego García, Manuel García García

**Affiliations:** 1 Department of Construction and Fabrication Engineering, National Distance Education University (UNED), 42005 Soria, Spain; sgallego118@alumno.uned.es; 2 Department of Construction and Fabrication Engineering, National Distance Education University (UNED), 28015 Madrid, Spain

**Keywords:** cybernetics, system dynamics, production management, maintenance management, Viable System Model, automotive industry

## Abstract

Designing, changing and adapting organizations to secure viability is challenging for manufacturing companies. Researchers often fail to holistically design or transform production systems. Reasons are often the conflict of interests between production and maintenance, the temporal divergence of their activities and their organizational structure. Thus, the aim of this study is to propose a holistic approach of how production and maintenance can be designed, changed or managed. Hereby, the Viable System Model was applied. This structure can be applied to any kind of structured organization and for its management with goals to be achieved in modern society; however, focus of the research is the coordination of production and maintenance management. The goal of the developed model is to be able to react to some potential production environments by taking coordinated decisions correctly and in the right moment based on the needed information. To ensure this, standardized communication channels were defined. In conclusion, this proposed approach enables production systems to have internal mechanisms to secure viability depending on all potential environment scenarios.

## 1. Introduction

The global logistics flows have increased dramatically in recent years due to a globalized economy that introduces inherent challenges to the establishment of international business [1] (p. 10). This evolution is combined with the demands of customers who want to be served with shorter delivery times [2] (p. 1) as well as with the increasing product variants in manufacturing and assembly processes that expose planning and control logistics to new challenges [3] (p. 797). The reasons are mainly the variability of final customers favored by competition activities. Other factors that generate uncertainty effects in the planning within the supply chain are, for example, minimum quantities in production as well as deviations of delivery lead times that make more difficult the production planning reducing the planning quality. The conventional answer to this challenge is to increase safety stocks to ensure the expected service level [4] (p. 179).

The success of the actors involved in a cooperative supply chain depends in an essential way on the extent to which they are able to deal with the dynamic market requirements [1] (p. 101)—together with efficient supply processes in costs, a reliable planning within the supply chain as well as robust production and logistics systems play a fundamental role for the long-term success of the actors within the supply chain. Robust production systems avoid negative effects on production processes by identifying potential disruptions and enabling an early adaptation [5] (p. 575). The result of a survey within a study of the German Association of Logistics ratifies this statement detailing that the reliability of logistics and production systems is the factor with greatest relevance considering logistics costs, reaction capabilities, flexibility and use of resources [6]. The logistics goal of robustness defined as the capacity of a system to deal with breakdowns, deviations and changes of the system environment without necessary changes in the structure or in production capacities [7] (p. 34) is gaining more and more importance as strategic feature to secure competitiveness.

Information in real time and its processing for production planning and control can give the correct answer how to face increasing dynamic requirements. However, the current planning and control logics do not provide the necessary support for this, which causes unexpected deviations in planning which cannot be compensated in the short term [7] (p. 160). The turbulences in production and in the supply chain create uncertainties that generate the need for coordination of planning processes, which has not been considered in a methodical way for the current concepts of production planning and control [8] (pp. 36–37). In this way, the insufficient collection and use of planning information lead to delays and deficits in the transmission of information that must be compensated through additional costs [9] (pp. 3–5). Moreover, due to the increasing automation and implementation of process control systems, more and more decentralized maintenance units are located next to production areas. Based on these facts, the common task for both production and maintenance must be developed to optimize the availability and condition of production plants to guarantee the satisfaction of the final customer. From a production point of view, maintenance has evolved from the “auxiliary need” to the holistic maintenance management, in which production and maintenance share a common goal [10] (p. 9).

According to a survey carried out by the VDMA (Mechanical Engineering Industry Association) organization of 240 European companies of all sizes and of different industries, in 2006, 78.5% of managers claimed that the importance of maintenance has increased significantly in recent years and 67.1% that it will continue to increase in the future [11] (p. 38). Considering this, it is prioritized between maintenance and production tasks. The negative long-term effects of insufficiently implemented maintenance measures are often accepted due to short-term production needs. Without denying the character of service, the role of maintenance is different if it is more widely understood as holistic maintenance management. Maintenance management is an essential component of production management but should not be used only to meet the production objectives (production of goods and services), but also the objectives of generating value, human objectives and environmental aspects of the company [10] (pp. 9–10).

Many approaches have been considered to make production and maintenance planning as efficient and flexible as possible. All pursue and many theoretically achieve the partial or total optimization of global production systems by making them efficient. However, when applying these theoretical internal logistics management models to practice, information systems should be used as a vehicle to communicate the necessary information to be managed within the organization. The reasons why the majority of applications of such concepts in information systems, as in ERP (Enterprise Resource Planning) systems, have failed are: the delay or lack of information, the misuse of production planning tools and maintenance, lack of coordination or conflict of interest between the production and maintenance departments, the non-consideration of the environment and the requirements of the final customer in a dynamic process of continuous improvement, leading all of them to take strategic, tactical or operational decisions at an optimal time or in an optimal way.

Research focus is the design and simulation of a coordination model for production and maintenance management using as a basis the current state of the art and pursuing high adaptability to environment changes. To achieve this, the Viable System Model (VSM) is used as a methodological structured approach and system dynamics is used to describe interrelationships between parameters and control loops in production and maintenance. The goal of the research is to generate an approach of how to design and develop production systems to be successful in all potential future environment scenarios considering developments in other areas of the company and in external fields of a company such as customer and supplier needs and conditions, politics, economy, technology, environment and energy regulations and society. In this context, simulation serves as tool to prove the approach in some potential future scenarios for production and maintenance coordination.

The initial hypothesis is that a production system built using the structure of the VSM will be able to react faster to environment changes and therefore to improve short-, medium- and long-term goals of every producing company.

## 2. Materials and Methods

This chapter contains a literature review of the viable system model, system dynamics as well as a description of the simulation technique and software employed.

### 2.1. The Viable System Model (VSM)

The Viable System Model (VSM), a cybernetic management model, was developed by Stafford Beer throughout his life [12] (p. 57). Beer deduced the VSM by taking the central nervous system of the human being and cybernetics as a basis to deal with complex systems. The VSM is built on three main principles: viability, recursivity and autonomy [13] (p. 434). The cybernetic model of every viable system consists always in a structure with five necessary and sufficient subsystems that are in relation in any organism or organization with the ability to conserve its identity with independency of its environment [12] (pp. 21–22).

To validate the research methodology, research and practical applications using the VSM were searched. Many authors have used the VSM as basis to describe and develop models how to deal with complex challenges in social and industry fields. Some of the topics worked and that give an indication of the scientific value of the approach are: organizational models for companies [14] (pp. 74–76), lean methods in terms of attenuating and amplifying variety [15], production system focused on “make-to-order” manufacturing [16], optimization of patient care in university hospitals [17], order booking process in mass production companies [18], production master program during launch processes [19], production planning in real time in the consumer goods industry [20] and integrated planning of distribution networks [21].

As described in the literature, the VSM is an unmatched conceptual and methodological tool for the modeling and design of organizations and its areas with the goal of being viable [22] (p. 16). Thus, the aim of the research is to propose a self-regulating approach of how designing and coordinating production and maintenance within manufacturing companies. For this reason, the Viable System Model is applied for this purpose.

### 2.2. System Dynamics (SD)

System dynamics is a computer-guided approach for studying, managing and solving complex feedback problems with focus on policy analysis and design [23] (p. 342). The origin of system dynamics [24] is the field developed by Forrester called “Industrial Dynamics” [25]. It proposes a methodology for the simulation of dynamic models by studying the characteristics of the information feedback of industrial systems.

SD has been applied to a great set of systems from corporate strategy to the dynamics of diabetes as well as for the cold war arms race between the USA and USSR. System dynamics can be applied to any dynamic system, with any time and spatial scale [24] (pp. 41–42). In a firm context, SD addresses three important issues: it helps to determine which policies should be used to control the behavior of the firm over time and, when the circumstances change, how these policies should be designed in order to have a robust response against change and how the organization can design its information feedback structure to assure the correct implementation of effective policies [26] (p. 3). The use of System Dynamics Modeling in Supply Chain Management has re-emerged in recent years after a long-stagnated period [23] (p. 342). The application of systems dynamics for the coordination of production management and maintenance makes sense since the cause–effect relationships show the interrelationships between the elements of the system and help to evaluate the influences of the different decisions in the global system. Therefore, it is selected for the research purpose.

### 2.3. Simulation Software

According to “VDI-Richtlinie”, simulation is “the reproduction of a system with its dynamic processes in an experimental model capable to gain knowledge that can be transferred to reality” [27] (p. 48). Simulation models are mainly used to support decision-making because they show the dynamic behavior of a system [28] (p. 28). Simulation is the only practical way to test models because our mental models are dynamically deficient, omitting feedback, time delays, accumulations and nonlinearities [24] (p. 37). For all these reasons, simulation is used to reproduce the conceptual model and to validate initial hypotheses. In the market, there are different software packages that enable system dynamics modeling such as: AnyLogic, DYNAMO, iTHINK, POWERSIM, STELLA and VENSIM [29] (p. 43). From all of them, VENSIM simulation software was selected for the research work. VENSIM is a registered trademark of Ventana Systems Inc., Harvard, MA, USA) serves as platform to build stock and flow model diagrams as well as causal loop diagrams. VENSIM also provides very powerful tools for analysis and validation of results and model structure as well as to determine most convenient policy options.

## 3. Literature Review on Production and Maintenance Management

This chapter provides the basic terminology needed to successfully perform this work.

### 3.1. Production Management

**Figure 1:**
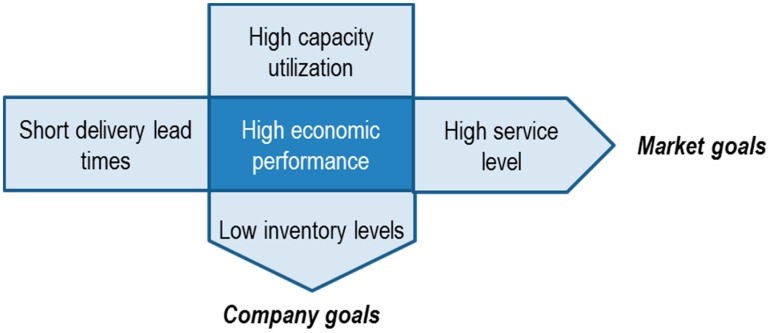
Multi-dilemma of production planning [32] (p. 36).

Production is the foundation of human activity. Natural resources are transformed into useful products through production processes to meet the needs of society [30] (p. 319). Production management contains the tasks of design, planning, monitoring and control of the productive system and business resources such as people, machines, material and information [31] (pp. 249–273). The multi-dilemma of production planning originates discussions repeatedly in the context of divergent objectives. This conflict of goals is shown in Figure 1 [32] (p. 36).

To analyze the tasks of production management, the Aachener Production Planning and Control (PPC) model, which is a reference model for its analysis, evaluation and design, is used [33] (p. 29).

**Figure 2:**
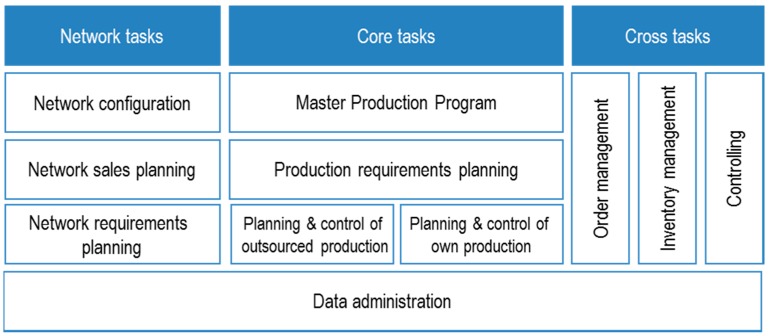
Production management tasks acc. to the Aachener PPC [33] (p. 30).

All tasks are distinguished vertically in Figure 2 according to their strategic, tactical or operational nature [33] (pp. 30–32). In the past, the focus was on operational and tactical problems; however, to successfully manage logistics in the future, an active level of strategic planning is also required [34] (p. 1).

### 3.2. Maintenance Management

**Figure 3:**
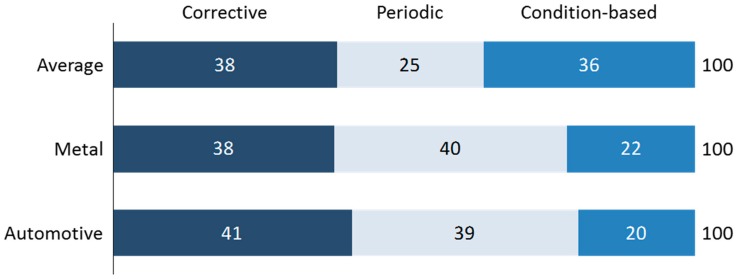
Survey result of 240 European companies on maintenance strategies [11] (p. 41).

Industrial maintenance is defined according to DIN (German Institute for Standardization) 31051 as the “combination of all technical, administrative and management measures during the life cycle of an observation unit in order to maintain the functional status or restoring it so that it can fulfill the required function” [35] (p. 26). The greater degree of complexity of technologies, systems and processes has considerably increased the demands on operating time. The increase of maintenance importance is a logical consequence since maintenance processes affect the quality, delivery time and costs and therefore company’s performance [15] (pp. 16–17). An optimal strategy must be found between the costs of preventive maintenance and the costs of machine failures [35] (p. 25). The basic maintenance strategies are corrective, periodic, preventive oriented to the condition of the object and predictive [35] (p. 104). The three “classic” maintenance strategies are still justified as seen in Figure 3.

The “ideal maintenance organization” does not exist [35] (p. 65) but depends on optimally combining the organizational forms with their advantages and disadvantages for the respective company [11] (p. 23).

According to experts, German companies spend around 140,000 million euros annually on maintenance of machines and installations. This, together with the fact that between 75% and 80% of the execution time of a maintenance order are activities without added value, defines a great potential for savings in production companies. Many of the time losses are in the interfaces between the different areas. The management of maintenance processes must ensure the change from a functional orientation to an orientation towards processes [35] (p. 60). For this purpose, tasks of maintenance management are: planning, control, analysis and measures execution [36] (p. 173).

### 3.3. Coordination Concepts of Production and Maintenance Management

Maintenance management of production units affects not only the maintenance personnel but also all the employees of the whole company [35] (p. 104). In the last two decades, there has been a general rethinking of industrial maintenance philosophy, from the maintenance of functions to a philosophy of value creation. However, for many maintenance tasks, there is a lack of adequate methods and instruments as well as information technology solutions [11] (p. 69).

Logistics can be described as an interdisciplinary strategy to optimize production. Availability is, therefore, a key factor for logistics and is also the link between logistics and industrial maintenance [35] (pp. 4–6). Maintenance should be “integrated” and dependent on all the functional areas involved in the added value [36] (pp. 9–10). This maintenance concept refers to the function and not just to the maintenance department within an organization. This is characterized by a distribution of the maintenance function “on several shoulders” [11] (p. 38). For all the above, some of the approaches to integrate or manage maintenance within the productive system are [36] (pp. 4–9):

- Total Productive Maintenance (TPM),
- Lean Maintenance,
- Total Lifecycle Cost Strategy (TLC),
- Reliability Centered Maintenance (RCM),
- Knowledge Based Maintenance.

## 4. Results

Within the research work, a conceptual model is developed and simulated for the integrated planning of production and maintenance based on the Viable System Model (VSM).

### 4.1. Conceptual Model Design Applying the VSM

#### 4.1.1. Methodology

This chapter describes the components of an integrated planning model for production and maintenance management. In a first step, the planning tasks are presented according to its planning level (strategic, tactical, and operational). Later, the recursion levels of the VSM are described. Then, the planning tasks are associated with the different recursion levels and the information flows are defined between recursion levels and systems of the VSM.

#### 4.1.2. Production and Maintenance Management Tasks Acc. to Their Planning Horizons

**Figure 4:**
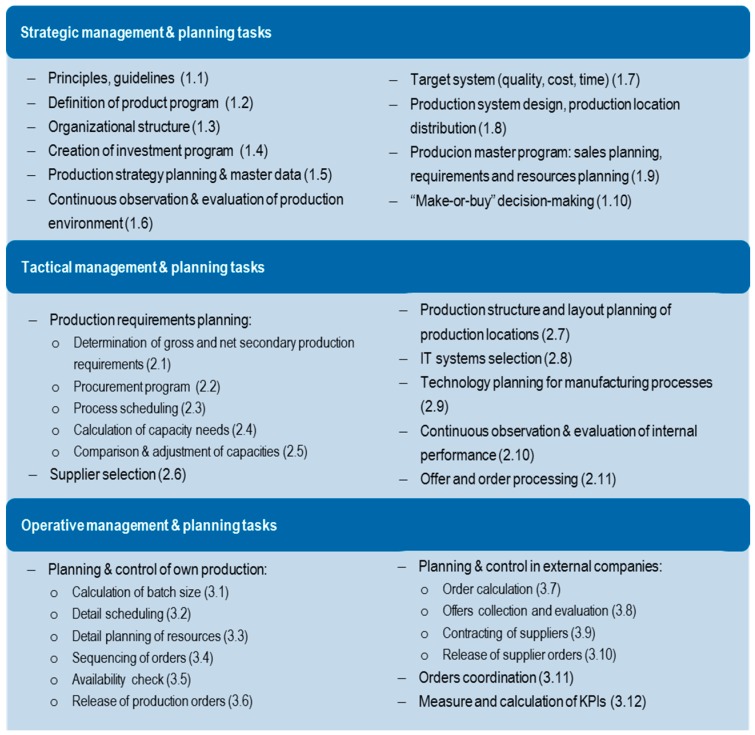
Production management and planning tasks acc. to time horizons (own elaboration).

**Figure 5:**
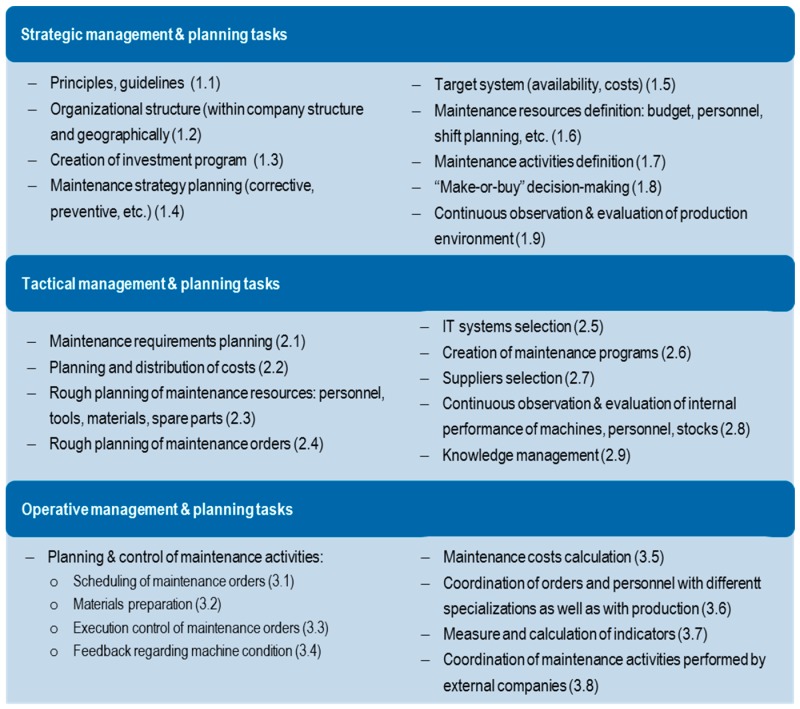
Maintenance management and planning tasks acc. to time horizons (own elaboration).

Production systems are considered important in relation to aspects of quality, time and costs [37] (p. 1). As explained before, planning tasks can be classified into strategic, tactical and operational planning depending on the respective planning horizon. Therefore, this classification was performed for the production management tasks in Figure 4. Moreover, with the increase of automation levels and the decrease of personnel in production, the importance of maintenance is increasing. With the same methodology, maintenance management tasks are also classified into the different time horizons in Figure 5:

#### 4.1.3. Recursion Levels Definition

**Figure 6:**
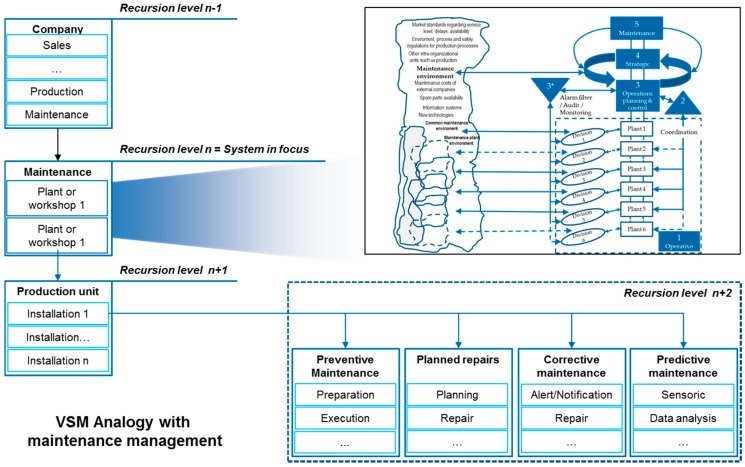
VSM recursion levels for company, production and maintenance (own elaboration).

A company is assumed as a viable system that is the first level of recursion in which the five systems neededto ensure viability are found. Therefore, in this research work, four levels of recursion can be differentiated as it is shown in Figure 6:

- The highest level, company (n − 1),
- The recursion level of production (n). In the same recursion level it can be found finance, human resources, IT, research and development, etc.
  ○ The recursion level of plant or production workshop, for example production management activities in an automotive production or assembly shop (n + 1),
  ○ The level of machine group, machine or installation with the associated activities for the different production activities such as preparation of the machine, change of tools, operation, control of production, etc. (n + 2),
- The recursion level of maintenance (n). In the same recursion level can be found finance, human resources, production, etc.
  ○ The recursion level of plant or production workshop—for example, maintenance management activities in an automotive production or assembly shop (n + 1),
  ○ The recursion level of installation or machine with the associated activities for the different maintenance activities: preventive, planned repairs, corrective, etc. (n + 2).

The activities in the recursion level n + 2 are no longer viable systems in contrast to the higher recursion levels because they do not contain a structure like that of the VSM, since they are the elements of production or maintenance execution.

Within this first level of recursion, company, the different functions of a company can be found, such as production, maintenance, commercial, finance, research and development, information systems, etc. In this research project, production and maintenance tasks will be analyzed in detail, recursion level n, but also considering the function of system 2 at the company level, n − 1, whose function is to coordinate the different functional areas of a company.

**Figure 7:**
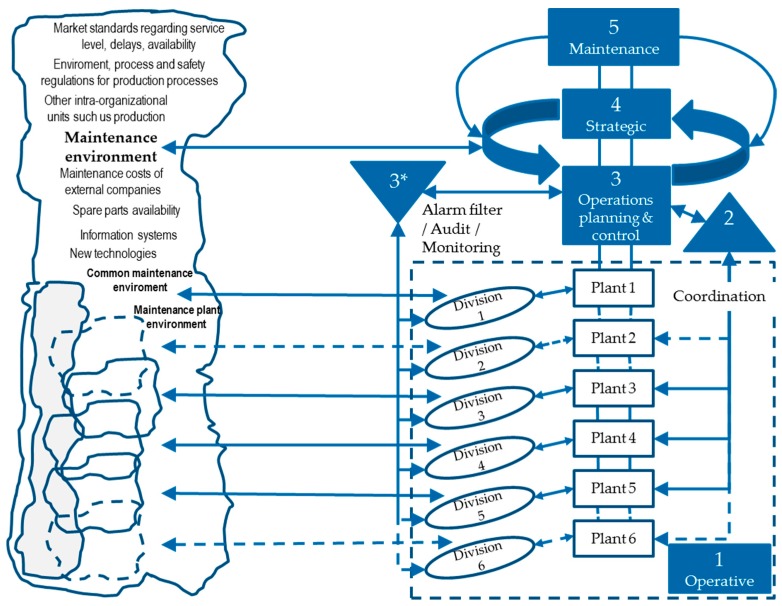
VSM analogy with maintenance management (own elaboration).

System 2 at company level plays the role of coordinator between the functional areas of the company trying to solve conflicts between them. Moreover, systems 1 at company level are all functional areas of every company such as production or maintenance. At the recursion level of production and maintenance (n), it is assumed that the different production plants or workshops will be the respective system 1, which also contains a viable system in each of these locations. The VSM of production and maintenance management within a company is described in the next paragraphs by the tasks performed by its five necessary systems:

- System 5 establishes the production and maintenance objectives and communicates them to the other management systems, systems 3 and 4.
- System 4 observes and collects essential information from the external environment of maintenance and production. Production environment is mainly represented by the demands of customers, but also by other factors such as information systems offered by the market for the management, planning and control of production, new manufacturing technologies and, in general, all factors affecting the production system such us market standards, delivery times, production strategies, delays, production costs in external companies for example to help in making decisions about outsourcing or to not manufacture certain parts or about the assembly of certain sets, etc. Maintenance environment could be described by the costs in the market for maintenance activities, average reaction times in the market as well as service level standards, information systems for maintenance, technology development, environmental and work safety regulations and customer delays. With these and other information from the external environment and information from system 5, system 4 creates a vision of what the production and maintenance areas have to be, and which should be the measures to be followed to reach that state. This vision is validated internally with system 3 so that system 4 makes the decision and system 3 makes the changes internally.
- System 3 is responsible for maintaining the internal stability of the model by optimizing the use of internal resources using the information received by system 4 about the clients as well as the information on the different divisions of system 1 through system 2. It would be related to functions such as operative production and maintenance management and control, information management, quality management, operative logistics planning and control, etc. Moreover, system 3* allows a quick response to possible emergencies in the manufacturing process or in the production control and monitoring by acting before information flows through system 2. It is capable of performing actions in real time if something happens outside of normal limits such as making changes in sequencing and production scheduling to avoid stopping production flow.
- System 2 is represented by the functions of coordination between the different production locations in daily activities. This system receives all the information of the different production plants and acts as a filter so that only the necessary information reaches system 3. The difference between both is in the time horizons of action. While system 2 performs functions in daily activities, the tactical system optimizes the performance of the internal system over a longer time horizon.
- System 1: each plant or workshop within the production system is an operational unit that includes the management of the unit and the division that performs the operational activities. An example could be an assembly workshop that contains the planning and production control departments responsible for the components of the workshop with their team leaders together with the operators that finally perform the production tasks.
- Environment: represents all the external factors that influence the production and maintenance management in a company. Figure 7 shows the environment of the entire maintenance area as well as of each plant or workshop.

Figure 7 was developed as a result of applying the VSM for maintenance management. In the same way it was done for production management following the descriptions of the previous paragraphs:

#### 4.1.4. Association of Planning Tasks to Recursion Levels

Production and maintenance management tasks were assigned to the VSM systems at recursion levels n and n + 1 defined before. In Table 1, the strategic production management tasks are classified and the tactical maintenance tasks and its classification to the VSM systems are shown in Table 2. It was done for all other production and maintenance tasks in the same way:

#### 4.1.5. Identification of Information Flows between Recursion Levels

Current technical literature agrees that the connection interfaces between recursion levels is extremely important [38] (p. 59). The goal is to determine basic links that can be transferred to any VSM in any company. The intensity of this connection between the levels varies according to the company [38] (p. 59). An exchange of information within the company and between levels of recursion is necessary to control the corporate environment, which generally has more information than can be processed in the company [14] (p. 287). Between the recursion levels, the following communication flows for both production and maintenance can be found:

- Between the company environment and system 4 at the production/maintenance recursion level,
- Between systems 5 of company and production/maintenance,
- Between systems 4 of company and production/maintenance,
- Between systems 3 of company and production/maintenance,
- Between systems 2 of company and production/maintenance,
- Between the operating units, systems 1, of company and production / maintenance,
- Between the alarm/monitoring filter (System 3*) of the company’s recursion level and system 4 of production/maintenance.

Between the two normative systems of company and production/maintenance, there is a flow of information that defines the degree of freedom of decision-making in which production/maintenance recursion level can act. Specifically, it means that the decisions taken by the management of the company are communicated to production and maintenance management defining its guidelines for autonomous decision-making within the respective areas. These guidelines can be financial, on personnel, on affectation to other areas, etc. In the same way, the objective levels such as production in term, production quality and production costs and adaptation capacity are influenced by decisions from the management, defining the priorities and the limits for the coordination among production areas. An example could be: the direction of the company in its strategic plan establishes the target production volume for the following years as well as the required flexibility in percentage on the production as well as the decrease in target costs. Of course, these decisions would influence the decision-making framework for production and maintenance that should adapt their methods and tools to be able to optimize costs, times and quality based on that flexibility also securing the required availability and adapting its maintenance strategy.

As explained during the research work, basic communication flows were defined. In total, 88 information connections were defined for the production recursion level specifying if the communication goes from company’s recursion level to production recursion level or between systems in production recursion level. Moreover, for the recursion level of maintenance, a total number of 77 information connections were defined specifying if the communication goes from company’s recursion level to maintenance recursion level or between systems in production recursion level. An extract is shown in Table 3 for the communication flows of the production recursion level:

### 4.2. Applying the Conceptual Model to an OEM Production System

The automotive supply chain is composed of suppliers at three levels (Tier 1–3), OEM (car manufacturers), distributors. For the work, we are going to focus on the production process of an OEM [39] (pp. 23–32):

1. Press shop
2. Bodywork shop
3. Paint shop
4. Engines shop
5. Assembly shops
6. Final assembly shops

Efficient logistics management is becoming a reality to survive in the automotive sector. The fragmentation and segmentation of vehicle models (such as hatchbacks, sedans, vans and pick-ups, cross-over coupes, roadsters, two-seater vehicles, SUVs, etc.) are growing. The complexity of customized models and variants is on the rise, especially as regards the way individual vehicles are equipped. The key trend in automotive production is the standardization of construction modules in common platforms. The modules refer to groups of components and related systems. The diversity of models is an important sales argument and delivery time is the key factor for the automotive market and in the manufacturing process. These requirements involve a change in assembly operations that need to be more flexible and agile [40] (pp. 24–25).

In the automotive industry, there are two approaches depending on when to start the assembly process. In the current market, most OEM producers use both to a greater or lesser extent:

1. Build-to-forecast: the assembly process starts before you have a customer for that car configuration.
2. Build-to-order: the assembly process is started only when there is a customer associated with that variant that is to be assembled.

On the other hand, and regarding the importance of maintenance in the automotive industry, it can be foreseen that, with the increase of automation and mechanization in the automotive industries, the production processes are becoming very sensitive to the machines and the human factor. Consequently, the role of equipment maintenance in the automotive industry as a mean to control and reduce costs while achieving the highest standards of reliability plays a fundamental role [41] (p. 514).

### 4.3. Model Simulation Using System Dynamics for an OEM Production System

In this step, the conceptual model for a manufacturing process is specified. To do this, the interrelations between indicators are defined by Causal Loop Diagrams (CLD). Later, the concepts are modeled in the VENSIM commercial simulation program. The simulation models present a simplified depiction of a real production and maintenance process to quantify the effect of decisions in a production system. Moreover, it contains the validation of the models and the presentation of the results obtained. Finally, the main conclusions are summarizedas well as a critical reflection of the work including a perspective for future research activities.

#### 4.3.1. Casual Loop Diagrams (CLD)

**Figure 8:**
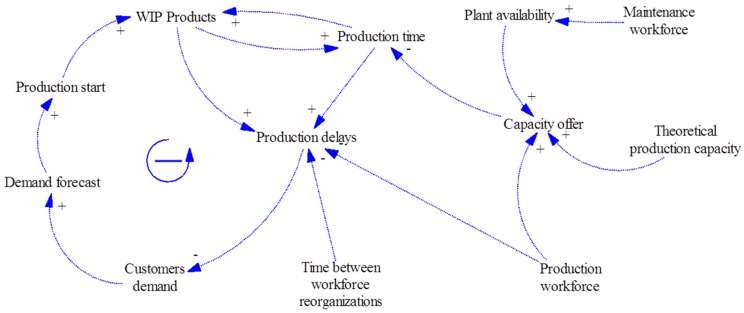
CLD for the production system.

The possibility of developing simulation models for production and maintenance management starts from the knowledge of the interrelations between its elements. Using casual loop diagrams (CLD), the effects of the changes of certain factors in their dependent parameters are shown. Five CLDs are developed from production management to maintenance planning. Figure 8 shows the CLD for the production system as an example:

#### 4.3.2. Methodology, Assumptions and Comparison Conditions

The aim of the simulation is to observe the impact of the delay in decision-making in a chain of manufacturing processes from the steel stamping process to the end of assembly in the automotive industry. The hypothesis is that a simulation model applying the VSM will present better results in terms of study parameters compared to the one that does not apply the VSM or that has delays in internal decision-making. The methodology for the simulation design is:

1. Definition of the objective, hypothesis and methodology
2. Number of simulation models
3. Simplification of the complexity of the conceptual model through assumptions
4. Criteria that make possible a comparison between models
5. Definition of quantitative parameters to obtain results and compare models
6. Definition of the production flow
7. Creation of the model based on the CLD (Casual Loop Diagrams)
8. Validation of the behavior of the simulation models
9. Determination of scenarios, simulation and extraction of results
10. Evaluation of the results and derivation of conclusions

First, assumptions are defined to simplify the model with focus on the simulation goal:

- Production times for car models are not variable
- Times for material transport and employee movements are not variable
- Distribution of finished products as given
- Procurement of raw material as given
- Demand does not change if customer service is better or worse
- Each order has a production unit
- Bill of materials are not considered

Some elements are equal in all models to make possible a comparison between the models under the same conditions:

- Same demand, same patterns of demand, demand replication
- Same production capacities per production step
- Same breakdowns per production step
- Same reaction times depending on the location
- Same task execution times
- Same workforce capacity
- The same maintenance capacity is considered for the same class of worker

#### 4.3.3. Key Performance Indicators (KPIs) for the Simulation Model

The objectives can be qualitative or quantitative. The research goal is to study the behavior of the different models in different situations of demand and configuration of maintenance and production areas. The results are quantified to evaluate the response according to the following key performance indicators:

1. Production on time (% per week)
2. Total production (# thous. cars)
3. Total downtime (days for all workshops)
4. Availability of final assembly workshop (%)
5. Capacity utilization of final assembly workshop (%)
6. Cumulated stocks (# Mill. cars)
7. MAD (Mean Absolute Deviation) (# thous. cars)
8. Cumulated demand (# thous. cars)

#### 4.3.4. Definition of the Production Flow in the Simulation Model

**Figure 9:**
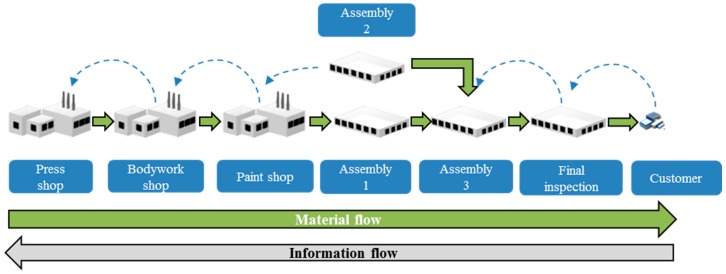
Simulation production flow (own elaboration).

The production process consists of a process from steel stamping to the final revision shop. The plants in the process are shown in Figure 9:

- Press shop
- Bodywork shop
- Paint shop
- Pre-assembly shop—assembly 1
- Mechanical assembly shop—assembly 2
- Final assembly shop—assembly 3
- Final inspection shop

All of them are in different shops and have production and maintenance units associated to each one of them.

#### 4.3.5. Design of the Intra-Organizational Simulation Model for Production and Maintenance Management

**Figure 10:**
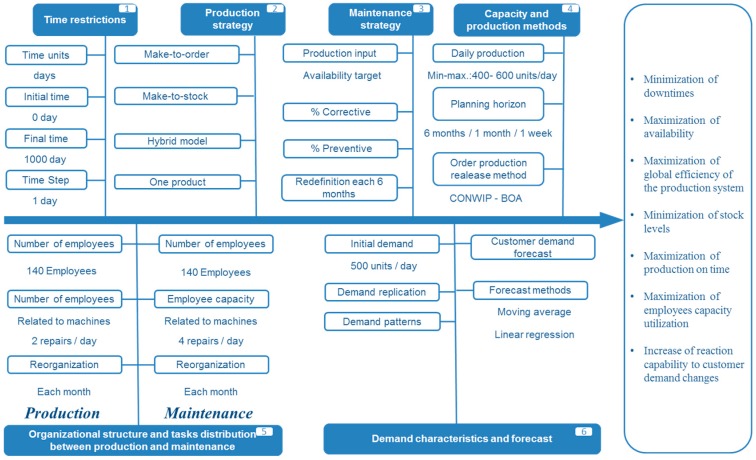
Ishikawa Diagram of Hypothesis for the simulation model (own elaboration).

As shown in Figure 10 the model is designed according to the following criteria:

1. Time restrictions: first, the modeler must define a time horizon and units of time. It is easy to fulfill that step by asking to what extent the simulation should be considered. In the case of the study, it has been decided to simulate four working years to evaluate influences in the medium and long term.
2. Production strategy: the model offers the possibility to manufacture according to the push or pull principle. A mixed strategy can also be chosen. It assumes the existence of a single product or model in the production flow.
3. Maintenance strategy: the simulation allows defining a preventive maintenance ratio so that it reduces the corrective according to a factor that is assumed.
4. Capacity and production methods: in the model there are two methods: CONWIP (CONstant Work in Progress) in which the quantity of products in production process is always the same and BOA (Workload-dependent order release) that initiates production of an order based on the current and expected workload for the plants.
5. Organizational structure and tasks distribution between production and maintenance: the simulation allows personnel changes in the areas of maintenance and production as well as between them. In addition, assumptions about the influence of the changes are made, for example, if there are more maintenance personnel, it will take less time to perform preventive maintenance tasks.
6. Demand characteristics and forecast: the model makes different forecasts using two different methods: moving average and linear regression.

#### 4.3.6. Simulation Model Validation

The validation of the simulation models can be done using different methods. In this process, some simulation variables will be used to observe their behavior and to evaluate if the models will be validated. Sterman defined 12 possible methods to validate system dynamics models [42] (p. 6). One of them, the test of extreme values, is used to validate the simulation model that shows that the response of the model is plausible when taking extreme values for different input parameters. For all models, the same input and output variables are chosen to analyze and validate the models. These input variables are the total number of employees, the initial stock in work in progress (WIP) and the production strategy, make-to-order vs. make-to-stock.

- For a lower number of employees, production on time (%) must be lower and the total stock should be higher because the production facility provides more products than employees can process. Moreover, utilization of shop capacities should be lower and therefore also the production output.

**Figure 11:**
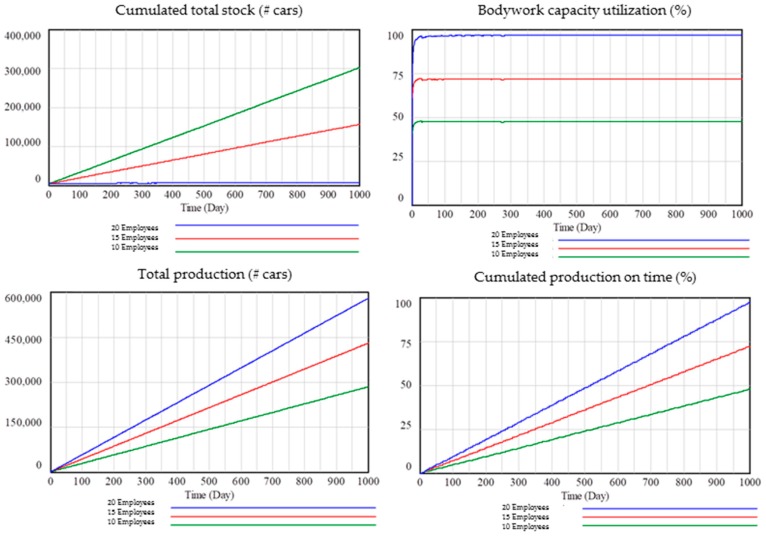
Validation with extreme values: number of employees.

As shown in Figure 11, it can be observed how the model behaves as expected. With 20 employees of maintenance and production per shop, the results for production on time (%) are 25% higher than with 15 employees and 50% higher than with 10 employees. In addition, total production and capacity utilization are higher for 20 employees than for 15 or 10 employees. Finally, cumulated stock over time is higher for 10 employees than for 15 and 20 employees because, with a lower number of production and maintenance employees, production volumes decreased.

- For higher initial stocks in WIP (work in progress) at initial time, production on time (%) must be higher and total stock should be higher in the first weeks because the production facility has more products within production process at the beginning. Moreover, utilization of shop capacities should be higher and therefore also the production output in the first weeks. Afterwards, due to the CONWIP method, the production WIP converges to a CONWIP goal that is equal to one week of demand and therefore all other indicators also converge.

**Figure 12:**
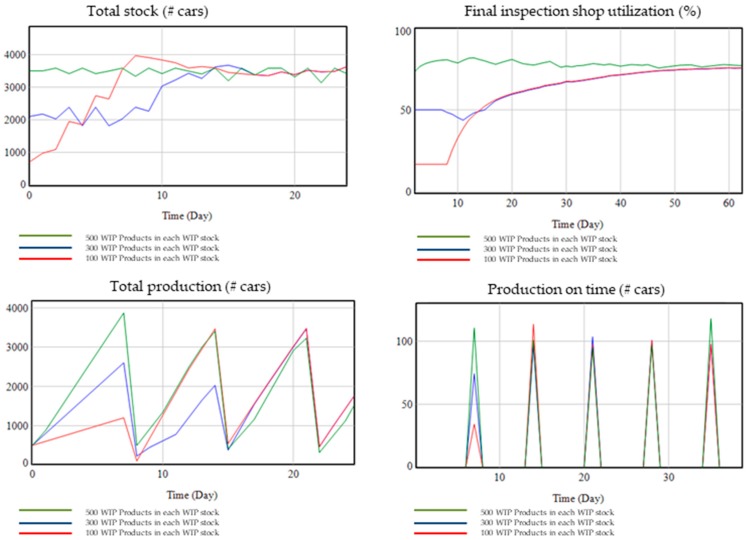
Validation with extreme values: WIP Initial stock.

In Figure 12, it is shown the model results for 500, 300 and 100 WIP products as initial WIP stocks in all intermediary stocks. The results show how initially the total stock, capacity utilization, production on time and total production at the beginning are higher for 500 WIP products at initial WIP; however, after 20–40 days, all of these performance indicators are equal for all set-ups of the initial WIP stock because the model initiates production of more products if the initial stock is low due to the CONWIP method set-up.

- For a make-to-order production, it is expected to have less stock during the production process, less capacity utilization and production output as well as less production on time than with a make-to-stock production.

**Figure 13:**
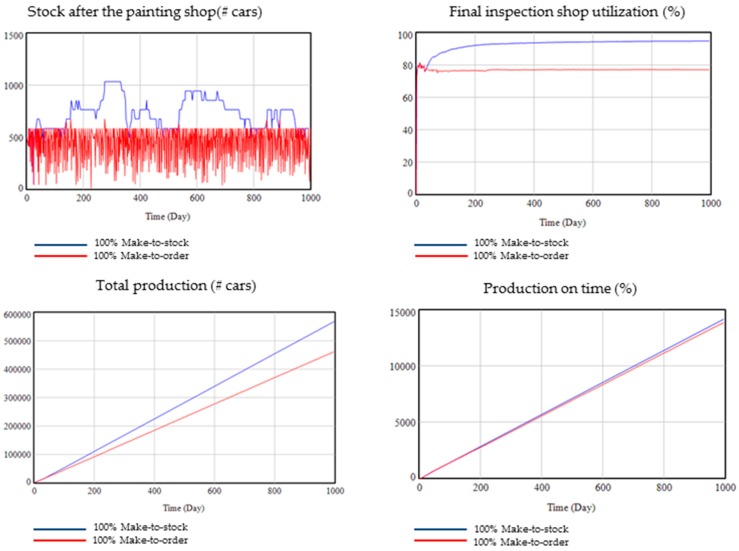
Validation with extreme values: Make-to-order vs. Make-to-stock.

As shown in Figure 13, it can be seen how the model results are as expected. In a make-to-order production stock, the stock after painting is lower than in make-to-stock production. Capacity utilization is 20% lower for make-to-order than for make-to-stock. Total production is higher for make-to-stock than for make-to-order because, in this configuration, only the customer orders are produced and if these orders are lower than the capacity, the extra-capacity is not utilized. Moreover, in a make-to-stock production, more products are served on time.

The model is validated because logical expected values are obtained for three different input parameters influencing multiple key performance indicators of the simulation.

#### 4.3.7. Scenario Definitions

Four case studies are proposed in which two different model configurations are simulated, trying to reflect the importance of decision-making. The VSM model takes earlier these decisions and therefore can benefit earlier from the new production system configuration:

1. Case study 1—reorganization of production and maintenance employees: the production area transfers workers to the maintenance area when there is a low customer demand. With this scenario, how the utilization of production workforce for maintenance activities could increase global production performance, if the decision is made in an early moment when demand starts to be lower than production capacities, is studied.
2. Case study 2—reorganization of production and maintenance employees: the maintenance area transfers workers to production when there is a high customer demand. With this scenario, how the utilization of maintenance employees in production activities could increase global production performance, if the decision is made in an early moment when demand starts to be almost at the maximum level of production capacities, is analyzed.
3. Case study 3—comparison between the CONWIP and BOA methods: for the same customer demand, the order initiation method varies. CONWIP (CONstant Work in Progress) initiates production of new orders maintaining the same WIP cars while BOA initiates production of new orders depending on current and future equipment workload. BOA tries to optimize utilization and CONWIP in the model tries to maintain a week of production demand always as WIP.
4. Case study 4—comparison between preventive and corrective maintenance: for the same demand, a low level of preventive maintenance activities and a high level of maintenance activities is simulated. With this scenario, the effect of the preventive level of maintenance activities can be simulated. In the models, a parametrization is done considering that preventive activities require a certain employee capacity but reduced the unexpected downtimes of the production shops.

#### 4.3.8. Simulation Results

For the four scenarios, the following results are obtained:

1. Case study 1—reorganization of production and maintenance employees with a low demand:
2. Case study 2—reorganization of production & maintenance employees with a high demand:
3. Case study 3—comparison between the CONWIP and BOA methods:
4. Case study 4—comparison between preventive and corrective maintenance:

## 5. Interpretation of the Simulation Results

1. Case study 1: the production area transfers workers to the maintenance area when there is a low demand for production. This decision is only taken in the VSM simulation model and moves three employees of a total of 20 employees from each production area to its respective maintenance area. The logic reason for this change is, since production employees cannot be fully utilized, they can help the maintenance units to perform maintenance activities in order to improve the production system performance. As it can be seen in Table 4, downtimes are more than 30% lower and production on time is almost 1% higher.
2. Case study 2: the maintenance area transfers workers to production when there is a high production demand. In this case, the Non-VSM model presents in one case 20 maintenance and production workers per plant or workshop while the VSM model changes to 23 workers for production and 17 for maintenance. Therefore, maintenance activities have less capacity while production capacity is increased. As it can be seen in Table 5, the VSM model presents downtimes almost 30% lower, total production is higher by 2000 cars, production on time is 0.2% higher and stocks are 26% lower.
3. Case study 3: it can be observed in Table 6 how the BOA method has better results in terms of quantity produced by almost 5%, in terms of production on time by 0.8% and in terms of capacity utilization by 4.3% since it starts the production of orders according to the workload. However, the BOA method has more cumulated stocks, 17% more than the CONWIP method, because orders are more time in production flow while securing capacity utilization. Therefore, in a VSM model of a manufacturing company, it can be decided whether to use BOA or CONWIP depending on company goals.
4. Case study 4: in the simulation model, there is a parameter that indicates the level of intensity of preventive maintenance. As it can be seen in Table 7, when making the decision of increasing preventive maintenance, availability increased by 3.7% and unexpected downtimes decreased by 39%.

Based on the results presented, it can be said that the VSM simulation model improves relevant KPIs when making the decision to move employees and can help production systems when deciding which production methods should be used as well as deciding the optimal percentage of preventive maintenance to be performed.

## 6. Conclusions

After completion of the research work, the importance of the design and management of a productive system that integrates the planning tasks of production and maintenance of a company has been proved. In addition, the following points can be successfully concluded:

- Identification of the objectives, key performance indicators and functions of production and maintenance management as well as basic methods and tools
- Clear evidence of the need for new approaches in the coordination of production and maintenance management after reviewing the state of the art
- Confirmation of the need for new communication and coordination systems as well as integral management of internal logistics as one of the biggest potentials to deal with current and future challenges in the manufacturing sector
- The application of the structure of the Viable System Model (VSM) for the design of the conceptual model for production and maintenance management provided a structure that has allowed the definition of information flows, planning levels as well as mechanisms of autonomy and escalation between levels within a company and within the areas of production and maintenance.
- System Dynamics has provided the notation and functions necessary for the design of simulation models using VENSIM as commercial software.
- The comparison of the different models for the four cases presented has shown the relevance of the organizational structure decisions and methods for production and maintenance management. In addition, it was proved how production and maintenance performance can be optimized by making decisions at the right moment. Key performance indicators such as total production, service level, downtime, etc. improved significantly when moving employees, changing production method or the maintenance strategy.

According to the results of the comparison, it has been demonstrated that correct and early decision-making through the application of the VSM gives a better response of all the key performance indicators. A similar response of the models without application of the VSM can occur when there are no external or internal changes that affect the productive system, that is, if the production system would be in a static situation. As environments and companies are always dynamic, the usefulness of the VSM as a principle for the definition of responsibilities, interfaces, escalation methods and decision-making are confirmed.

In conclusion, this proposed approach can increase the efficiency of internal logistics models such as the productive system in its interaction with industrial maintenance. Finally, it is important to point out new ways of research or new ways to continue improving the project carried out:

- Improve the conceptual model with all the different methods and tools by level of recursion and task as well as its inclusion in the simulation model
- Extend the study for all internal logistics processes to generate a tool to help management, since, as we have seen, the markets require flexibility and right now the companies do not have mechanisms to make the right decisions in most of the cases. Many of them centralize maintenance units and a few years later they decentralize it again, which suggests that there is no strategic or tactical direction.
- Apply the simulation model based on the VSM for a real productive system

## Figures and Tables

**Table 1:** Strategic production management tasks and its classification to VSM systems (own elaboration).

Key Performance Indicator	Production Recursion Level			Plant Recursion Level		
Systems of the VSM	S5	S4	S3	S5	S4	S3
Principles, guidelines (1.1)	X	-	-	X	-	-
Definition of product program (1.2)	X	-	-	-	-	-
Organizational structure (1.3)	-	X	-	-	-	-
Creation of investment program (1.4)	-	X	-	-	-	-
Production strategy planning & master data (1.5)	-	X	-	-	-	-
Continuous observation & evaluation of production environment (1.6)	-	X	-	-	-	-
Target system (quality, cost, time) (1.7)	-	X	-	-	-	-
Production system design, production location distribution (1.8)	-	X	-	-	-	-
Production master program: sales planning, requirements and resources planning (1.9)	-	X	-	-	-	-
“Make-or-buy” decisions (1.10)	-	X	-	-	-	-

**Table 2:** Tactical maintenance management tasks and its classification to VSM systems (own elaboration).

Key Performance Indicator	Maintenance Recursion Level			Plant Recursion Level		
Systems of the VSM	S5	S4	S3	S5	S4	S3
Maintenance requirements planning (2.1)	-	-	X	-	-	-
Planning and distribution of costs (2.2)	-	-	-	-	-	X
Rough planning of maintenance resources: personnel, tools, materials, spare parts (2.3)	-	X	-	-	-	-
Rough planning of maintenance orders (2.4)	-	X	-	-	-	-
IT systems selection (2.5)	-	-	-	-	X	-
Creation of maintenance programs (2.6)	-	X	-	-	-	-
Suppliers selection (2.7)	-	-	X	-	-	-
Continuous observation & evaluation of internal performance of machines, personnel, stocks (2.8)	-	-	-	-	-	X
Knowledge management (2.9)	-	-	-	-	X	-

**Table 3:** Extract of information flows in the production recursion level (own elaboration).

No.	Information on the Production Recursion Level	From … to …
1	Information about stopped facilities	From System 1 to 2/3
...	...	...
28	Number of orders and quantity produced	From System 1 to 4/5
29	Number of orders that have met the required deadlines and quantity	From System 1 to 4/5
30	Average Delivery time of the products	From System 1 to 4/5
31	Number of defective Deliveries	From System 1 to 4/5
32	Number of Deliveries with claims	From System 1 to 4/5
33	Total number of changes made to production schedules	From System 1 to 4/5
...	...	...
88	Information about the economic environment of a productive plant	From environment to 4/5

**Table 4:** Case study 1—Results.

KPI-No.	Key Performance Indicator	Without Reorg. of Employees (non-VSM)	With Reorg. of Employees (VSM)
1	Production on time (% per week)	98.4	99.2
2	Total production (# thous. cars)	458	458
3	Total downtime (days for all workshops)	139	97
4	Availability of final inspection shop (%)	94.1	94.1
5	Capacity utilization of final inspection shop (%)	76.3	76.3
6	Cumulated stocks (# Mill. cars)	3.3	3.3
7	MAD (# thous. cars)	23	23
8	Cumulated demand (# thous. cars)	458	458

**Table 5:** Case study 2—Results.

KPI-No.	Key Performance Indicator	Without Reorg. of Employees (non-VSM)	With Reorg. of Employees (VSM)
1	Production on time (% per week)	96.9	97.1
2	Total production (# thous. cars)	579	581
3	Total downtime (days for all workshops)	143	100
4	Availability of final inspection shop (%)	94.2	94.2
5	Capacity utilization of final inspection shop (%)	96.5	96.8
6	Cumulated stocks (# Mill. cars)	5.7	4.2
7	MAD (# thous. cars)	12.3	12.3
8	Cumulated demand (# thous. cars)	582	582

**Table 6:** Case study 3—Results.

KPI-No.	Key Performance Indicator	CONWIP	BOA
1	Production on time (% per week)	96.3	97.1
2	Total production (# thous. cars)	542	568
3	Total downtime (days for all workshops)	143	100
4	Availability of final inspection shop (%)	94.2	94.2
5	Capacity utilization of final inspection shop (%)	90.4	94.7
6	Cumulated stocks (# Mill. cars)	4.6	5.4
7	MAD (# thous. cars)	12.3	12.3
8	Cumulated demand (# thous. cars)	577	577

**Table 7:** Case study 4—Results.

KPI-No.	Key Performance Indicator	10% Preventive	100% Preventive
1	Production on time (% per week)	97.1	97.1
2	Total production (# thous. cars)	579	579
3	Total downtime (days for all workshops)	192	117
4	Availability of final inspection shop (%)	91.3	95.0
5	Capacity utilization of final inspection shop (%)	96.5	96.5
6	Cumulated stocks (# Mill. cars)	5.1	4.9
7	MAD (# thous. cars)	12.3	12.3
8	Cumulated demand (# thous. cars)	579	579
